# Object-Based Reliable Visual Navigation for Mobile Robot

**DOI:** 10.3390/s22062387

**Published:** 2022-03-20

**Authors:** Fan Wang, Chaofan Zhang, Wen Zhang, Cuiyun Fang, Yingwei Xia, Yong Liu, Hao Dong

**Affiliations:** 1 Anhui Institute of Optics and Fine Mechanics, Hefei Institutes of Physical Science, Chinese Academy of Sciences, Hefei 230031, China; wanfan8@mail.ustc.edu.cn (F.W.); fangcy@mail.ustc.edu.cn (C.F.); xiayw@aiofm.ac.cn (Y.X.); liuyong@aiofm.ac.cn (Y.L.); 2 Science Island Branch of Graduate School, University of Science and Technology of China, Hefei 230026, China; 3 China National Tobacco Quality Supervision Test Center, Zhengzhou 450001, China; dongh@ztri.com.cn

**Keywords:** topological path planning, visual navigation, object-level topological semantic map, Bernstein polynomial

## Abstract

Visual navigation is of vital importance for autonomous mobile robots. Most existing practical perception-aware based visual navigation methods generally require prior-constructed precise metric maps, and learning-based methods rely on large training to improve their generality. To improve the reliability of visual navigation, in this paper, we propose a novel object-level topological visual navigation method. Firstly, a lightweight object-level topological semantic map is constructed to release the dependence on the precise metric map, where the semantic associations between objects are stored via graph memory and topological organization is performed. Then, we propose an object-based heuristic graph search method to select the global topological path with the optimal and shortest characteristics. Furthermore, to reduce the global cumulative error, a global path segmentation strategy is proposed to divide the global topological path on the basis of active visual perception and object guidance. Finally, to achieve adaptive smooth trajectory generation, a Bernstein polynomial-based smooth trajectory refinement method is proposed by transforming trajectory generation into a nonlinear planning problem, achieving smooth multi-segment continuous navigation. Experimental results demonstrate the feasibility and efficiency of our method on both simulation and real-world scenarios. The proposed method also obtains better navigation success rate (SR) and success weighted by inverse path length (SPL) than the state-of-the-art methods.

## 1. Introduction

Over the last few decades, autonomous mobile robots have gained increasing attention for various applications, such as indoor service, surveillance missions, and search-and-rescue. Safe and reliable autonomous navigation is of crucial importance for mobile robots to execute their main tasks in complex environments. Vision-based navigation has become a popular research area due to the richness and practicality of vision sensors [1]. Most existing practical indoor vision navigation methods focused on path planning with a prior precise metric map, such as an occupancy grid map [2] and dense map [3]. Generally, these maps are constructed with Simultaneous Localization And Mapping (SLAM) algorithms, which can perform well in a conditional ideal environment [4]. In spite of their remarkable results, some challenging environments, such as unstructured indoor areas and dynamic objects, pose great challenges for the performance of visual navigation methods. With the development of deep learning, the learning-based visual navigation methods demonstrate strong navigation performance to the above problems [5,6,7], while they need a large number of training datasets to improve generalization capabilities. Therefore, it is necessary to exploit a reliable and feasible visual navigation method.

In general, the performance of practical map-based visual navigation is mainly affected by the variations of the environment [8]. Many state-of-the-art algorithms [9,10,11,12] demonstrate that robust object feature detection is significant for improving the reliability of visual navigation. In [9,10,11], based on the precise metric map, they improved the reliability of path planning by fusing semantic object information to achieve safe and efficient visual navigation. Li Tang et al. present a topological local-metric framework, which achieves long-term autonomous navigation through the fusion of object-based topological associations and local metric information [12]. Thus, to achieve reliable visual navigation, in this paper, we propose a novel object-based topological path planning method, unlike the above approach, which tightly connects objects through a semantic topological graph structure and performs reliable global path planning based on the semantic guidance of the objects. The proposed method mainly includes two issues: effective global path searching and feasible trajectory generation.

On the one hand, for global path searching, an object-constrained topological path-searching method is proposed. Firstly, a lightweight topological semantic map is constructed to reduce the dependence of visual navigation on a high-precision map, by modifying our previous work [13] with the graph memory and topological associations. Then, based on the constructed map, an object-based heuristic graph search method is proposed to effectively search global topological paths. We extract the highly-dimensional semantic-geometric features of objects based on 3D object detection and use the multi-attribute constraints on the feature to provide heuristic evaluation for graph search. What’s more, a robot-centric relative topological association constraint is proposed to provide weights for graph search in the absence of global poses. With the presented method, an optimal and shortest global path is obtained, which improves the reliability of the visual navigation system.

On the other hand, for trajectory generation, to reduce the effect of the global cumulative error on visual navigation, we propose a novel segmented smooth trajectory generation and refinement method based on object guidance and Bernstein polynomial parameterization. In [14,15], it is also confirmed that segmented trajectories can improve the accuracy of path planning. Inspired by this, we convert the trajectory generation problem into a nonlinear programming problem. First, an active visual perception and object guidance strategy is proposed to achieve the effective segmentation of global paths. Then, the trajectory of segmentation is represented based on the multi-constraint property of Bernstein polynomials. Based on the proposed method, we can obtain smooth and dynamically feasible trajectories, and the reliability of the proposed visual navigation method is further improved.

**Figure 1:**
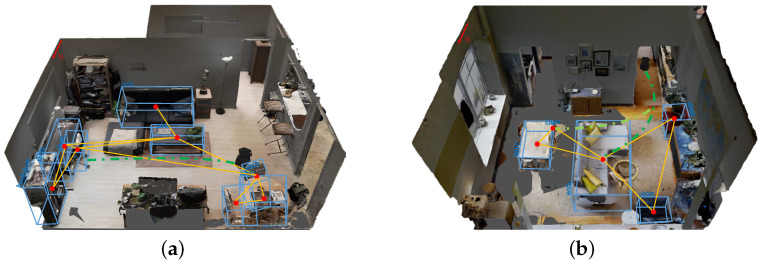
Illustration of the proposed object-level topological path planning for visual navigation. (**a**,**b**) shows two different indoor scenarios. The blue boxes represent the 3D object detection of object-level landmarks. The red dots indicate the nodes of the topological map. The yellow lines indicate the edges of the topological map. The green curve is the feasible navigation trajectory generated based on the proposed method.

The illustration of the proposed object-level topological path planning for visual navigation is shown in Figure 1. The main contributions are summarized as follows:

- A novel object-based visual navigation method is proposed, where an object-constrained topological path-searching method is proposed for the first time to significantly release the dependence on a precise metric map and improve the reliability of visual navigation.
- A segmented smooth trajectory generation and refinement method is proposed, based on the object guidance and Bernstein polynomial parameterization. We implement adaptive smooth trajectory generation to further improve the effectiveness and efficiency of global path planning.
- Experimental results on both simulation and real-world scenarios validate the feasibility and efficiency of our methods.

## 2. Related Work

In this section, we briefly review visual navigation methods from the views of navigation map representation, path searching, and trajectory optimization.

### 2.1. Navigation Map Representation

For navigation map representation, classical metric-based navigation map representations are well established by accurately encoding the 3D geometric information of the environment. Multiple representations have been constructed by different methods, for example, in [16,17,18,19,20], they represent maps as precise and sparse 3D landmarks by specific visual features (such as point feature and line feature). However, they cannot perform global path planning and obstacle avoidance tasks. In contrast, dense representations attempt to provide high-resolution models of the 3D geometry [3]. Available sensors also facilitate the construction of highly accurate geometric maps, such as occupancy grid maps [2]. Although these models are more suitable for obstacle avoidance and path planning for mobile robots, they also typically require the storage of large amounts of data, and the high-precision maps are not easy to maintain and scale [5]. Other works try to improve the reliability of map representation by embedding semantic information [19,20,21,22]. Currently, topology maps are widely explored to represent the environment in abstract graphs, achieving a simple and compact lightweight representation [23,24,25]. However, the pure topological solution is not suitable for robot navigation that requires metric guidance [12]. Thus, some work constructs topological representations that are highly consistent with the metric map [26] or embeds local metric information [12]. Although it makes the map lightweight, it also limits the high scalability of topological maps. Moreover, due to the widespread successful application of deep learning, the learning-based map representation approach has also attracted a lot of interest. Some topological methods work with human-like exploration of pre-established topological maps [6,7]. It relies on a large number of labeled datasets and cannot be applied well to an unfamiliar environment. Other approaches build topological representations that incorporate semantics [27]. Inspired by the above work, our work focuses on making visual navigation lightweight, scalable, robust, and efficient. We intend to achieve this by integrating the high-dimensional semantic and geometric information of objects into the structure of the graph, which enables reliable navigation capabilities.

### 2.2. Path Searching

The topological representation of the environment already provides a discrete working space for path planning. Most works that perform path search in this type of workspace are also referred to as C-space search. Depending on the way of discretizing the C-space search, path-searching methods can be subdivided into two predominant groups [28]: the sampling-based method and graph search method. Rapidly Random Tree (RRT) [29] is the most famous sampling-based path search method, which stimulates the growth of a tree, starting from a starting point and dynamically creating branches [30]. Later, lots of path-searching methods have been proposed by improving the RRT method, such as RRT-Connect [31], Heuristic RRT (RRT*) [32], Probabilistic Roadmap Method (PRM) [33], and Fast Marching Tree (FMT*) [34]. The sampling-based path-searching algorithm is asymptotically optimal, which means that the number of samples may be larger over time to approach the global optimal solution [35] and requires the use of larger memory resources to store all the samples. The graph search-based path search algorithm completely or partially accesses the constructed topology graph until it finds a path connecting the initial point and the goal point. The classical graph search-based path search algorithm is the Dijkstra algorithm [36]. Currently, several improved versions are obtained by speeding up the search, reducing the computational complexity, including A* [37], Dynamic A* [38], Lifelong Planning A* (LPA*) [39], and Hybrid A* [40]. In this paper, inspired by the A* algorithm, we propose an object-based heuristic path-searching method, which performs heuristic evaluation by semantic–geometric features of objects and weight constraints based on topological associations between objects.

### 2.3. Trajectory Generation

Generally speaking, the existing initially searched paths cannot be directly executed by the robot due to dynamic constraints and the inherently poor smoothness of the paths. Therefore, a control function is required to parameterize the paths, which generates smooth trajectories and adjusts them to the robot’s motion constraints. In the CHOMP proposed by Ratliff et al. [41], the optimal solution is approximated from the feasible path utilizing the gradient optimization technique. Van Den Berg et al. [42] optimized the obtained initial path by differential dynamic programming. In HOOP [43], quadratic programming is employed to transform the trajectory refinement into a higher-order segmented polynomial processing. In [44], they proposed a generative algorithm for minimizing snap that represents the trajectory as a piece-wise polynomial function. Savkin et al. [45] proposed a method for smooth trajectory generation based on curvature constraints. However, this method assumes that the obstacles are smooth and constrains the robot model, which makes it difficult to apply to real-world complex scenarios. In [46,47], Bernstein bases are used to perform trajectory optimization, and they directly generate safe and dynamically feasible trajectories, which confirms the feasibility of the algorithm. In this paper, we first perform the segmentation of global paths by active visual perception and object guidance. After that, the individual segmented paths are parameterized based on Bernstein polynomials to generate smooth and feasible trajectories via transforming the trajectory optimization problem into a nonlinear programming problem.

## 3. Overview of the Framework

In this paper, we propose an object-level topological path planning method that enables mobile robots to perform reliable visual navigation tasks in the absence of a precise navigation map. Inspired by the idea of human-like navigation, we allow the robot to observe its surroundings through vision sensors and estimate ego-motion based on semantic-geometric information about objects and association information between objects in the environment.

**Figure 2:**
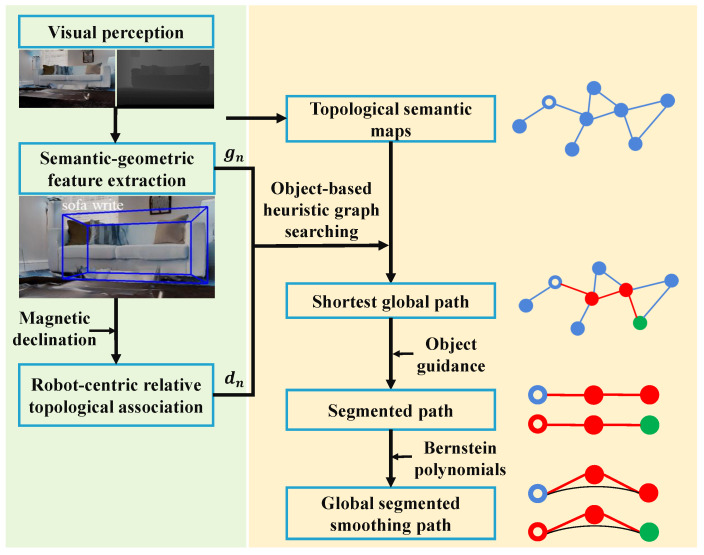
The block diagram outlines the specific modules in the proposed object-level topological path planning system and the connections between them.

The overall framework of the proposed object-level topological path planning is described in Figure 2. We take the outputs of the lightweight navigation mapping, global graph search, as well as trajectory generation and refinement modules to produce dynamically feasible global paths. The whole framework is composed of two sections. Firstly, an object-constrained topological path-searching method is applied to object-based visual navigation, which includes topological semantic mapping and object-based heuristic graph search, as described in Section 4. Topological semantic mapping provides a lightweight environment model for visual navigation (Section 4.1). Object-based heuristic graph search provides an optimal shortest initial path for visual navigation (Section 4.2). Secondly, on the one hand, a global path segmentation strategy based on active visual perception and object guidance is proposed to divide the extracted initial global topological path into multiple segments, which reduces the global cumulative error in navigation, as shown in Section 5.1. On the other hand, Bernstein basis [47] is used to generate smooth and dynamically feasible trajectories, which improves the effectiveness and efficiency of path planning (Section 5.2).

## 4. Object-Constrained Topological Global Path Searching

**Figure 3:**
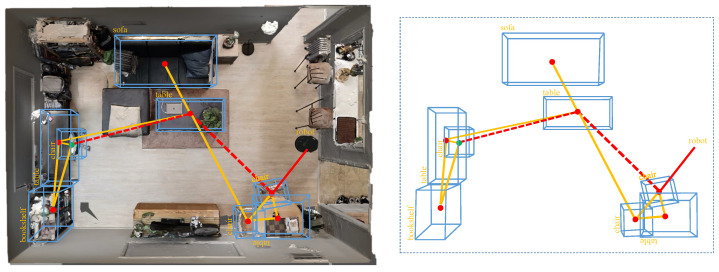
Illustration of the object-based heuristic graph search. The red dashed line indicates the shortest global topological path.

In this section, to extract the initial global path, we achieve the object-constrained topological global path searching via the lightweight object-level topological semantic mapping and an object-based heuristic graph search. In this paper, the overall path-searching cost is $\left(f_{n}=g_{n}+d_{n}\right)$. $g_{n}$ is a heuristic evaluation by matching the multi-attribute constraint based on the semantic–geometric feature of the goal object. $d_{n}$ is the relative topological association constraint of the topological edge. Finally, a global topological path is obtained, which is a unidirectional graph composed of adjacent topological edges concatenated together, as expressed in Equation (1). The illustration of the object-based heuristic graph search method is shown in Figure 3.

(1) $$\begin{array}{c} graph\\ \left(i<j<n\right) \end{array}=\left\{\begin{array}{c} node_{1},\ldots ,node_{i},\ldots ,node_{j},\ldots ,node_{n}\\ edge_{ij} \end{array}\right\}$$

### 4.1. Representation of the Object-Level Topological Semantic Map

**Figure 4:**
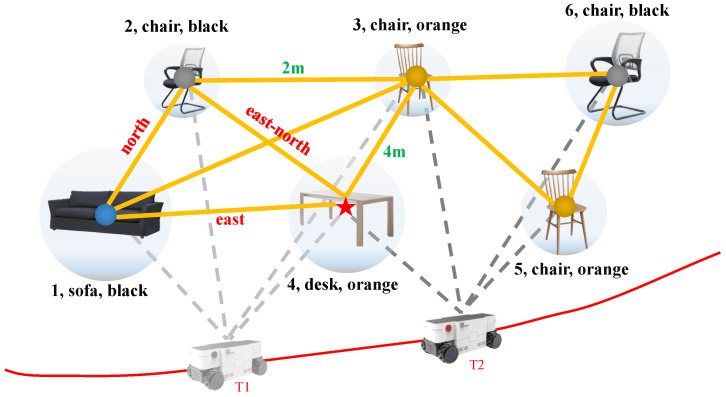
Illustration of our proposed agent-centric object-level topological semantic map.

The map is a fundamental representation of aspects of interest (e.g., landmarks, obstacles) describing the environment of robot operates [4]. In vision navigation, the robot needs to compute the position information of landmarks from arbitrary locations to assess the perceptual quality of candidate landmarks. Objects are the basic constituents of the environment. Inspired by the way humans navigate, we pay more attention to recent relative motion between objects other than the highly accurate global position for global navigation. Therefore, in this paper, referencing our previous work [13], we represent the environment by using a lightweight abstract topology graph that records the relative associations between objects. The basic structure of this map is a graph defined as $G=\left\{N,E\right\}$, where *N* and *E* denote the nodes and edges of the graph, respectively. The representation of the map is shown in the semantic scene sub-graph in Figure 4.

**Node Representation:** We take the objects in the scene as topological nodes and define the nodes with the semantic properties of the objects themselves, such as class and color. Thus, for each node $N_{i}$ belonging to *N*, the corresponding properties are defined as $N_{i}=\left\{ID,class,center,n_{i}\right\}$. ID is the serial number added to the graph in order. $center\left(x,y,z\right)$ is the coordinate of the object center point obtained by fusing deep information. $n_{i}$ is defined as a proxy for additional properties of the node. For example, color, functional and operational properties, 6D pose (position and orientation), etc. To achieve accurate matching between objects, different from traditional visual features, we propose a multi-attribute high-dimensional semantic-geometric feature for object-level nodes, which includes attributes such as category, color, and 3D geometric center. The multi-attribute high-dimensional semantic-geometric feature is obtained as described in Section 4.2.1.

**Edge Representation:** For topological edges, the associated object-level node relative relationship attributes are used to define, for example, the relative direction and distance between nodes. Thus, for each edge $E_{ij}$, connecting the neighboring nodes $N_{i}$ and $N_{j}$, belonging to *E*, the corresponding properties are defined as $E_{ij}=\left\{dis_{ij},yaw_{ij},e_{ij}\right\}$. $dis_{ij}$ is a rigid relative distance between node $N_{i}$ and $N_{j}$, which is also used as the weight of the topology graph. $yaw_{ij}$ is a relative direction between node $N_{i}$ and $N_{j}$ in the geomagnetic coordinate system. We introduce IMU to calculate the magnetic declination angle. $e_{ij}$ is defined as a proxy for additional properties of the edge.

### 4.2. Object-Based Heuristic Graph Searching

#### 4.2.1. Heuristic Evaluation Based on Semantic–Geometric Feature

**Figure 5:**
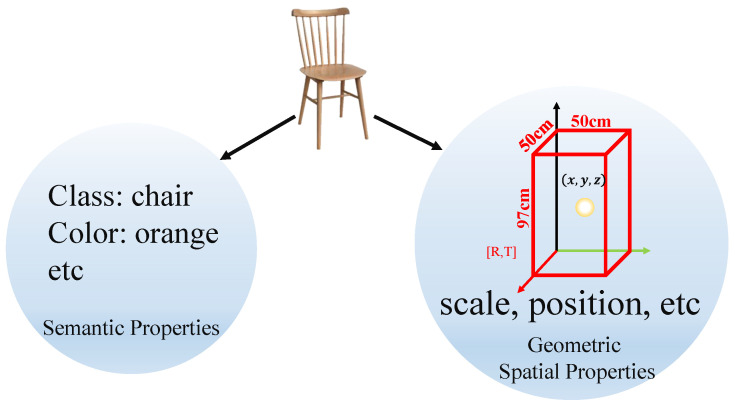
Schematic representation of the high-dimensional semantic–geometric features of the object. High-dimensional semantic-geometric features consist of semantic features (class, color, etc.) and geometric spatial features (scale, position, etc.).

In this section, to meet the requirements of object-based heuristic path searching, a highly robust semantic–geometric feature is constructed, which fuses the high-dimensional semantic properties of objects with geometric spatial properties. On the one hand, the multi-attribute constraint matching based on semantic–geometric features between objects can provide heuristic evaluation $\left(g_{n}\right)$ for path searching and improve the efficiency of path searching. On the other hand, semantic–geometric features of objects can effectively improve the robustness of visual navigation under scene changes, such as illumination and occlusion. The illustration is shown in Figure 5.

**Figure 6:**
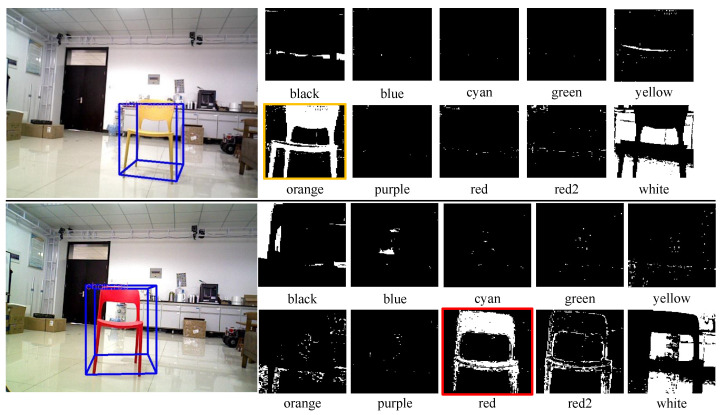
Experimental results of 3D object detection with embedded color attributes. The top and bottom columns represent the color feature extraction for orange and red chairs, respectively.

For the above reasons, we use VoteNet [48], the 3D object detection method that achieves advanced performance, to extract semantic information and geometric spatial features of the object (scale size, centroid coordinates, pose, etc.). VoteNet is proposed inspired by the Hough voting-based 2D object detector, which votes on virtual centroids of objects directly from a point cloud and generates a set of high-quality 3D object proposals by aggregating voting features [49]. Thus, it reaches the state-of-the-art performance on two large indoor benchmark datasets (ScanNet [50], SunRGBD [51]). In particular, to improve the differentiation of objects of the same class, we modified VoteNet by making it possible to output the color properties of objects directly. Firstly, we select the smallest outer rectangle of the object based on the projection of the 3D box onto the 2D image to extract the salient area of the object and eliminate the background interference. Then, we maintain color invariance by converting the original RGB space into a more robust HSV space [52]. Finally, we select the color with the largest area percentage as the object color property. The experimental results are shown in Figure 6.

**Figure 7:**
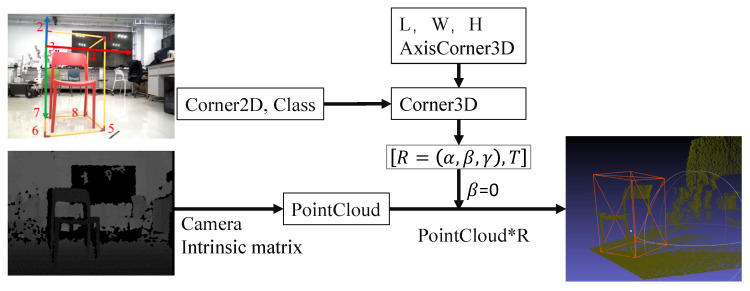
The block diagram of the proposed PNP-based annotation tool for 3D object detection.

In addition, to increase the detection accuracy of VoteNet in the real-world environment, we collect and add some data for training based on the data format of the SunRGBD dataset. Since there is no open-source annotation tool for the existing indoor dataset, we make a homemade tool for indoor 3D point cloud annotation inspired by the PNP approach [53]. The annotation process is shown in Figure 7. Marking is divided into three main steps. Firstly, a cube frame is surrounded by the road marker to match its scale before annotation. After that, a corner point of the cube frame is used as the origin of the world coordinate system. The world coordinates and pixel coordinates of the extracted corner points are converted to the cubic frame’s pose by PNP and used as the pose of the landmark. Finally, all the point cloud data are corrected to the horizon level. We will open-source the income of this tool soon.

**Figure 8:**
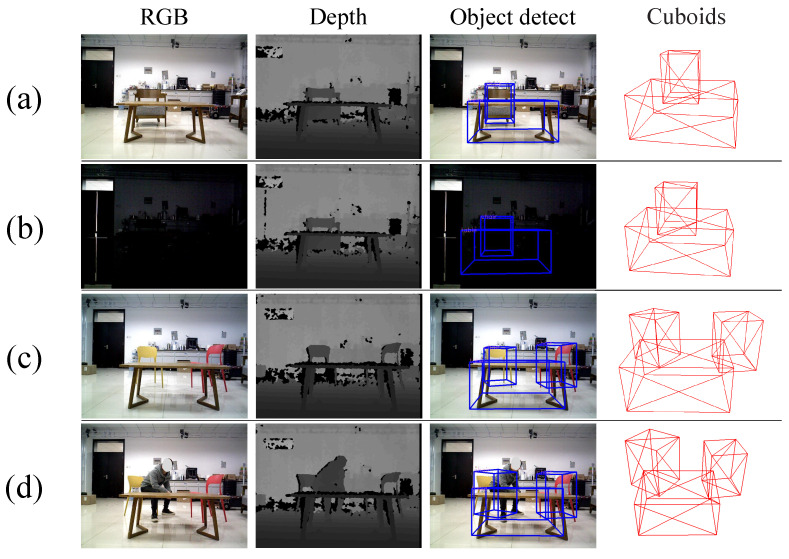
Four examples of 3D object detection results in real-world environments. (**a**,**b**) are the detection comparison results of the illumination variation. (**c**,**d**) are the comparison results of detection of occlusion and a dynamic object. Experimental results indicate that the proposed high-dimensional semantic-geometric features have high robustness.

The result of 3D object detection in the real-world environment is shown in Figure 8. The experimental results show that the 3D object detection method has high robustness.

#### 4.2.2. Robot-Centric Relative Topological Association Constraint

**Figure 9:**
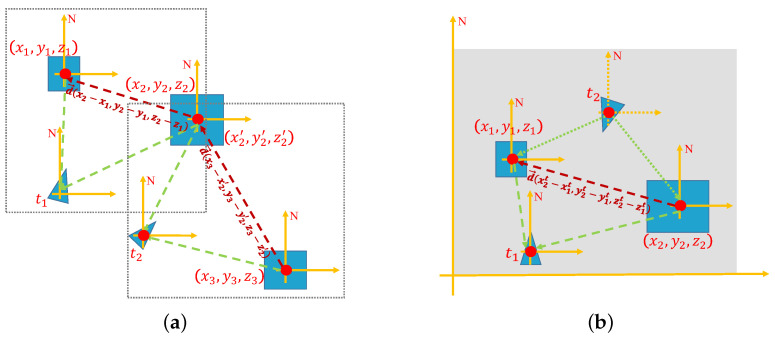
Illustration of the robot-centric relative topological association. (**a**) is the illustration of topological association information of the environment. (**b**) is the illustration of topological association based on the principle of coordinate invariance of vectors.

The high-dimensional semantic-geometric feature detection of objects has provided highly robust visual features for environment modeling and path searching. However, the semantic information learned by the model has not yet been considered relevant to autonomous robot movement. The information from object detection still needs to be translated into information in the robot coordinate system. To ensure effective path searching through the constructed topological map without global poses, we propose a robot-centric relative topological association constraint to obtain the weight of the shortest path searching. The topological association information of the environment is shown in Figure 9a. In the constructed topological map, we represent the relative directions between adjacent nodes as direction vectors $\vec{d}=\left(x_{2}-x_{1},y_{2}-y_{1},z_{2}-z_{1}\right)$ in the calculation by converting the landmark and robot rigid body coordinate as well as the robot rigid body coordinate and the geomagnetic coordinate. As shown in Figure 9b, since the direction of the geomagnetic coordinate system is usually constant, based on the principle of coordinate invariance of vectors, the relationship between adjacent fixed nodes does not change with time and space. The specific implementation of the pseudocode is shown in Algorithm 1. Finally, $d_{n}$ is obtained by calculating the Eulerian distance $\left(d_{n}=\sqrt{{\left(x_{2}-x_{1}\right)}^{2}+{\left(y_{2}-y_{1}\right)}^{2}+{\left(z_{2}-z_{1}\right)}^{2}}\right)$ of the topological edge.
**Algorithm** **1** Robot-Centric Relative Topological Association**Require:** Adjacent landmarks $N_{1}$ and $N_{2}$, given the coordinates $CN_{12}$ of $N_{1}$ in the camera coordinate system at the moment $t_{2}$, solve the coordinates $BN_{22}$ of $N_{2}$ in the robot coordinate system at the moment $t_{2}$;1:$CN_{11}=\left(x_{1},y_{1},z_{1}\right),CN_{21}=\left(x_{2},y_{2},z_{2}\right)\leftarrow$ The coordinates in the camera coordinate system at the moment $t_{1}$;2:$\left[R,T\right]$ Conversion matrix of the camera coordinate system to the robot rigid body coordinate system;3:$BN_{11}=R*CN_{11}+T,BN_{21}=R*CN_{21}+T,$$BN_{12}=R*CN_{12}+T$;4:$yaw_{1},yaw_{2}\leftarrow$ The deflection angles of the robot’s rigid body and magnet moments $t_{1}$ and $t_{2}$, respectively;5:$MN_{11},MN_{12},MN_{21}\leftarrow \;$$MN_{it}\cdot x=BN_{it}\cdot x*\cos\frac{yaw_{t}*\pi }{180}+BN_{it}\cdot y*\sin\frac{yaw_{t}*\pi }{180}$; $MN_{it}\cdot y=BN_{it}\cdot y*\cos\frac{yaw_{t}*\pi }{180}-BN_{it}\cdot x*\sin\frac{yaw_{t}*\pi }{180}$;6:$\vec{d}=\left(MN_{21}\cdot x_{2}-MN_{11}\cdot x_{1},MN_{21}\cdot y_{2}-MN_{11}\cdot y_{1}\right)$;7:${\mathrm{MN}}_{22}={\mathrm{MN}}_{12}+\vec{d}$;8:$BN_{22}\leftarrow {\mathrm{MN}}_{22},yaw_{2}$.

## 5. Object-Guided Topological Trajectory Generation and Refinement

To obtain smooth and feasible trajectories, in this section, the initial topological path is refined based on real-time object guidance and Bernstein polynomials. Firstly, the topological path is segmented based on the spatial information of the local objects detected in real time. Then, the segmented path is refined by parameterizing them with Bernstein polynomials to form a smooth trajectory as the final global trajectory.

### 5.1. Object-Guided Trajectory Segmentation and Refinement Strategy

**Figure 10:**
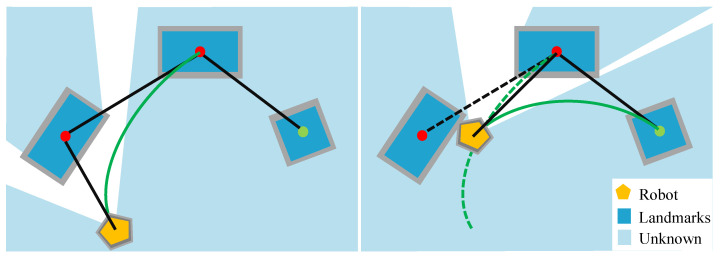
Illustrates of the object-guided topological trajectory generation and refinement.

**Figure 11:**
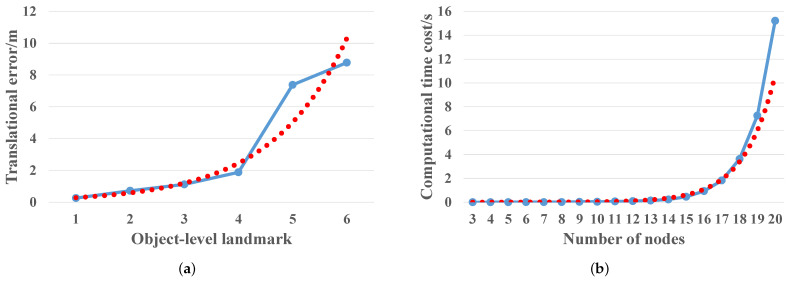
The performance of global segmentation strategy verification experiment. (**a**) Cumulative translation error. The solid blue line indicates the cumulative translation error of converting the six objects on the global topological path to the current robot coordinate system. The red dashed line indicates the exponential trend of the error. (**b**) Computational time cost. The solid blue line indicates the computational time cost of Bernstein polynomials as the number of nodes increases. The red dashed line indicates the exponential trend of the computational time cost.

To reduce the impact of global cumulative error, a global path segmentation strategy based on active visual perception and object guidance is proposed, which divides the initial global path into multiple segments to maintain the navigation error in the local area and reduce the computational complexity of the trajectory generation process. Firstly, as the navigation case illustrated in Figure 10, for a given global topological path, when the robot navigates in the map, the robot body is used as the starting point, and the active visual observation and the matched objects are used as the intermediate guidance point to guide the robot to the next unknown target point through positional permutation. The above process is executed cyclically until the goal location is navigated. In addition, we conducted several experiments to verify the effectiveness of the global segmentation strategy proposed above, as shown in Figure 11. On the one hand, we plot the variation curve of the global cumulative translation error of the goal object position with the increase of the number of landmarks on the global path, as shown in Figure 11a. The experimental results show that the cumulative translation error will have an exponential growth trend with the increase of the number of landmarks on the global path. Thus, the proposed global segmented path strategy is beneficial to eliminate the effect of global cumulative translation error on path planning. On the other hand, we plotted the variation curve of the computation time of the Bernstein polynomial with the increase of the number of nodes, as shown in Figure 11b. The experimental results show that the time cost of computation tends to increase exponentially with the increase in the number of nodes. Therefore, the segmentation of paths facilitates the execution of online global path planning.

**Figure 12:**
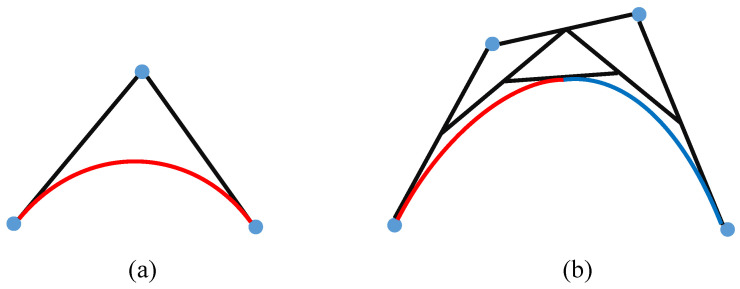
(**a**,**b**) are schematic diagrams of Bessel curves of second order and third order, respectively. (**b**) shows a geometric example of de Casteljau’s algorithm to split Bernstein polynomials. The Bessel curve on the left is shown in red, and the curve on the right is shown in blue.

Secondly, it is important to generate a smooth and dynamically feasible trajectory for robust and accurate autonomous navigation of the robot. Thus, in this paper, we propose a novel trajectory refinement strategy by turning global path planning into a nonlinear planning problem and using properties useful for Bernstein polynomials to accomplish these tasks. According to the Bernstein polynomial theorem, all continuous functions on the interval $\left[a,b\right]$ can be approximated by polynomials, as shown in Figure 12a. In the case of this paper, it is also known as a Bezier curve B(t), which has some special constraint properties [47]:

- **Endpoint constraint property**. The Bessel curve always connects the starting and ending control points in series without passing through any intermediate control points;
- **Convex hull constraint property**. The Bezier curve consists of a set of control points that are completely confined within a convex hull defined by its Bernstein coefficients;
- **De Casteljau algorithm constraint property**. The de Casteljau algorithm implements the decomposition of a Bernstein polynomial defined on an interval into multiple segments for computation. The illustration is shown in Figure 12b.

**Figure 13:**
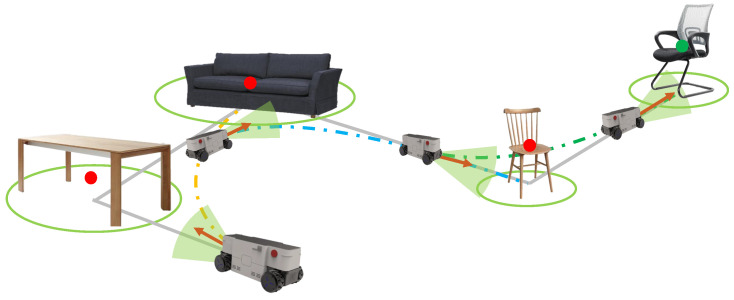
The schematic diagram of the proposed global segmented smooth trajectory generation. The gray solid line is the global topology shortest path. The three dashed lines (yellow, blue, and green) are the three trajectories generated online, respectively. Each segment of the trajectory is a Bessel curve generated by taking the robot’s rigid body as the starting control point and the first currently invisible node in the one-way diagram as the end control point in turn.

The schematic diagram of the proposed global segmented smooth trajectory generation is shown in Figure 13.

### 5.2. Bernstein Basis Segmental Trajectory Formulation

A two-dimensional, *n*th-order Bernstein polynomial, $B_{t}^{n}$, is defined as:

(2) $$B_{N}\left(t\right)=\sum_{i=0}^{n}p_{n}^{i}b_{n}^{i}\left(t\right),t\in \left[t_{0},t_{f}\right]$$

where $p_{n}^{i}\in R^{2},\left(i=0,\ldots ,n\right)$ is the set of control points of the *n*th piece of the Bezier curve. $b_{n}^{i}\left(t\right)$ is the Bernstein polynomial basis, which is defined as:

(3) $$b_{n}^{i}\left(t\right)=\left(\begin{array}{c} n\\ i \end{array}\right)\frac{{\left(t-t_{0}\right)}^{i}{\left(t_{f}-t\right)}^{n-i}}{{\left(t_{f}-t_{0}\right)}^{n}}$$

where $\left(\begin{array}{c} n\\ i \end{array}\right)=\frac{n!}{i!\left(n-i\right)!}$ is the binomial coefficient. Our online segmented trajectory generation is based on the end-point constraint property of the Bessel curve. The robot body is used as the first control point in each segment. Based on the order of the nodes in the one-way graph, the first node in the one-way diagram that is currently not detectable in the current local view range is used as the last control point, as shown in Figure 13. Therefore, according to the real-time local environment, the nodes in the one-way graph visible during navigation are used as current control points, and thus, the control points within each segment are dynamically variable, $p_{n,m}^{i}=\left\{p_{n,1}^{i},p_{n,2}^{i},\ldots ,p_{n,m}^{i}\right\}\in R^{2}$, *m* denotes *m*-segments.

According to the number of shortest path nodes and the number of visible range nodes, the set of *m*-segments can be expressed as shown in Equation (4):

(4) $$f_{B_{n}\left(t\right)}=\left\{\begin{array}{c} \sum_{i=0}^{n_{1}}p_{n_{1},1}^{i}\left(\begin{array}{c} n_{1}\\ i \end{array}\right)\frac{{\left(t-t_{0}\right)}^{i}{\left(t_{f}-t\right)}^{n_{1}-i}}{{\left(t_{f}-t_{0}\right)}^{n_{1}}},t\in \left[t_{0},t_{f}\right]\\ \sum_{i=0}^{n_{2}}p_{n_{2},1}^{i}\left(\begin{array}{c} n_{2}\\ i \end{array}\right)\frac{{\left(t-t_{0}\right)}^{i}{\left(t_{f}-t\right)}^{n_{2}-i}}{{\left(t_{f}-t_{0}\right)}^{n_{2}}},t\in \left[t_{0},t_{f}\right]\\ \vdots \\ \sum_{i=0}^{n_{m}}p_{n_{m},1}^{i}\left(\begin{array}{c} n_{m}\\ i \end{array}\right)\frac{{\left(t-t_{0}\right)}^{i}{\left(t_{f}-t\right)}^{n_{m}-i}}{{\left(t_{f}-t_{0}\right)}^{n_{m}}},t\in \left[t_{0},t_{f}\right] \end{array}\right.$$

Based on the de Casteljau algorithm constraint property, the overall formula can be expressed as:

(5) $$B_{n}\left(t\right)=\sum_{j=0}^{m}\sum_{i=0}^{n_{j}}p_{n_{j},j}^{i}b_{n_{j}}^{i}\left(t\right)$$

It is worth noting that when the last node is seen, it will become a one-order Bernstein polynomial.

## 6. Experiments

In this section, to evaluate the proposed method well, we perform a series of experiments on both simulation and real-world environments. We mainly compare our method with two types of navigation methods: a learning based navigation method and a classical metric-map based navigation method. All experiments have been run on a PC with Intel i7-8700K CPU 3.7GHz, NVIDIA GTX1080GPU, and 24GB RAM. The experimental video link is at https://github.com/CASHIPS-ComputerVision/Paper-videos (accessed on 2 March 2022).

### 6.1. Experimental Setup

#### 6.1.1. Multi-Constrained Local Path Planning Strategy

**Figure 14:**
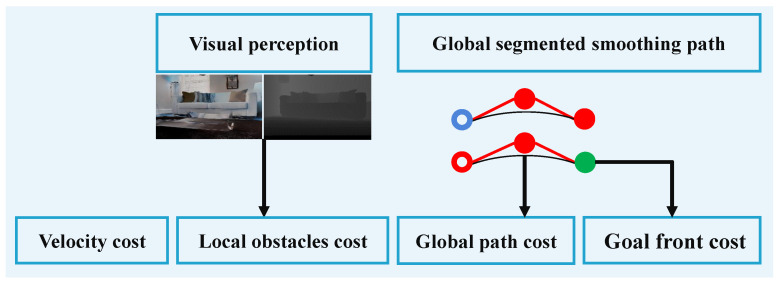
The block diagram outlines the specific modules in the proposed multi-constrained local path planning strategy.

To enable the proposed object-level topological path planning approach to perform practical navigation tasks, we first integrate a multi-constrained local path planning strategy into the overall framework to achieve local obstacle avoidance, as shown in Figure 14.

The multi-constrained local path planning strategy takes the endpoint of each global segmented trajectory as the stage goal point of local planning. To obtain the local linear and angular velocities, as shown in Equation (6), it determines the optimal local trajectory by calculating the overall loss cost $\left(C_{n}\right)$ of the sampled trajectory. The overall loss cost includes the global segmented Bessel trajectory cost constraint (Gdist), the velocity cost constraint $\left(\frac{1}{{\dot{x}}^{2}}\right)$, the pointing target cost constraint (Ddist), and the obstacle cost constraint (obs). The velocity cost constraint $\left(\frac{1}{{\dot{x}}^{2}}\right)$ is the translational component of the sampled velocity on the current trajectory, which keeps the robot’s velocity within a certain range.

(6) $$C_{n}=\alpha \mathrm{obs}+\beta \mathrm{Gdist}+\gamma \mathrm{Ddist}+\frac{\delta }{{\dot{x}}^{2}}$$

#### 6.1.2. Environment

**Figure 15:**
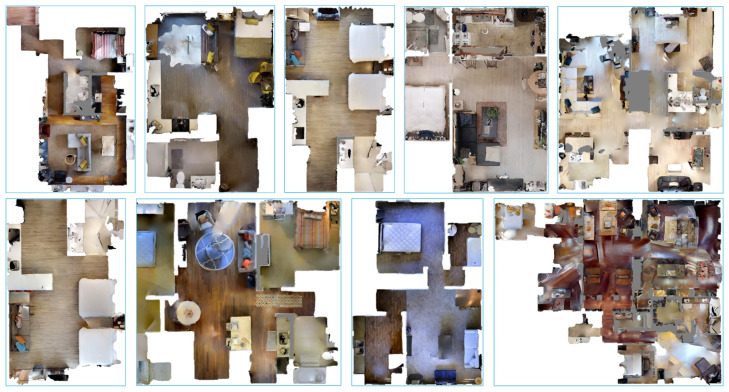
Top view of nine simulation experiment scenarios in the Gibson dataset.

**Figure 16:**
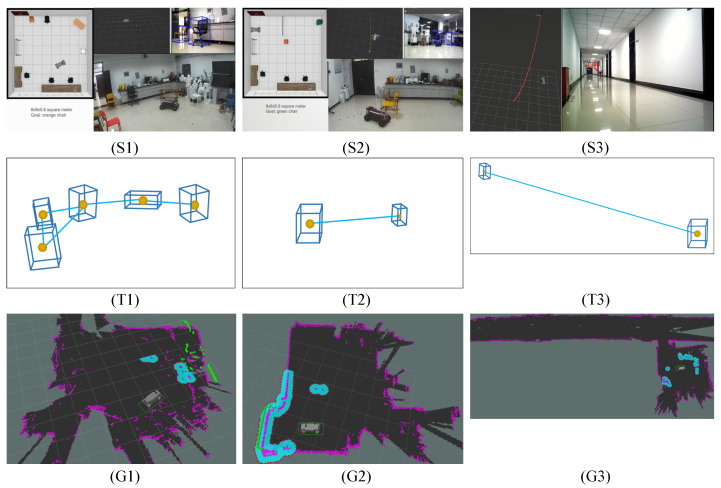
Example of navigation in the real world. (**S1**–**S3**) show the test scenes of our method in the real world. (**T1**–**T3**) correspond to the topological semantic maps of the three scenes, respectively, which are generated by our previous work [13]. (**G1**–**G3**) correspond to the high-precision occupancy grid maps of the three scenes, respectively, which are generated by the OGMADWA method.

**Figure 17:**
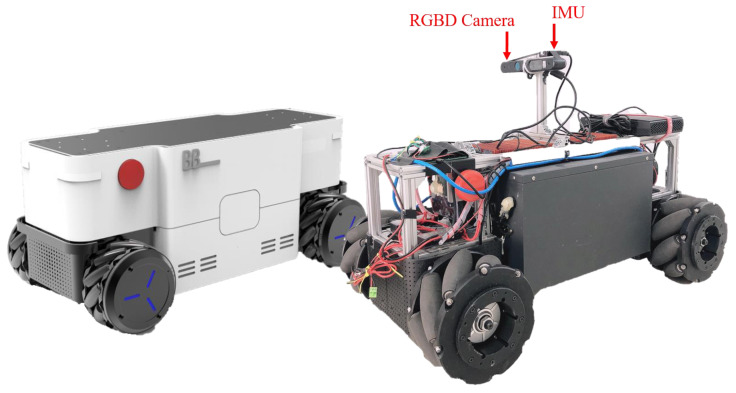
A four-wheeled mobile robot platform called BootBot. It is equipped with an Xtion RGBD camera and an inertial measurement unit (IMU). These sensors are placed 20 cm above the middle of the body.

- **Simulation Experiment Setup:** We build the simulation environment with the Gibson dataset [54]. The Gibson dataset is visually realistic, since it consists of reconstructions of real-world scenes [5]. As shown in Figure 15, we finally selected nine simulation scenes by excluding scenarios that contain multiple floors and empty rooms. In the simulation experiments, the baseline methods are the state-of-the-art learning-based navigation methods, including Neural Topological SLAM (NTS) [5], Active Neural SLAM (ANS) [6], and Metric Spatial Map + RL (MSMRL) [7]. All of these methods use RGBD camera settings. MSMRL is an end-to-end navigation method based on the local metric map constructed by geometric projections of depth images, and it performs navigation decisions using Reinforcement Learning (RL). ANS is a baseline that integrates metric map and learning-based navigation method to perform agent movement control. NTS models the environment as a topological map. However, different from our method in this paper, it performs navigation through retrieval image goals. Following the method in NTS [5], the test scenarios in this paper are classified as easy, medium, and difficult, depending on the distance between the start and end locations, which are: Easy (1.5–3 m), Medium (3–5 m), and Hard (5–10 m).
- **Real-World Experiment Setup:** As shown in S1, S2, and S3 of Figure 16, in the real-world experiment, we choose two typical indoor environments to evaluate our method, including weakly textured corridors and offices. During the experiments, we set up three navigation tasks with different difficulties, named Test 1, Test 2, and Test 3, as shown in Figure 16. Test 1 is continuous navigation with no obstacles and has multiple landmarks. Test 2 is a more challenging navigation with dynamic obstacles that do not exist in the constructed map. Test 3 is the long-distance navigation for large scenarios, which is the most challenging task for most existing navigation methods. We use a four-wheeled mobile robot to record RGB-D images and inertial measurement unit (IMU) measurements, as shown in Figure 17. It is equipped with an Xtion RGBD camera and an inertial measurement unit (IMU). The RGBD camera returns a regular RGB image and a depth image that is used for real-time semantic landmark detection. The IMU returns high-frequency inertial guidance data for magnetic declination detection.
  In the real-world experiment, to further evaluate the proposed navigation method, we compare our method to a classical navigation method (called OGMADWA), which is combined by a high-precision Occupancy Grid Map [7], the global path-searching method A* [37], and the local path planning method DWA [55].

#### 6.1.3. Evaluation Metrics

In this paper, we use the navigation Success Rate (SR) and Success weighted by inverse Path Length (SPL) as the evaluation metrics to compare the performance of our method with other methods. SPL is one of the most important metrics for evaluating navigation performance, which considers the effectiveness and efficiency of the agent in accomplishing the navigation task. It is worth noting that only when the motion of the mobile robot in the state space is continuous, the experiment is determined effectively. For a success rate, we set the “arrived” signal, and only if the robot takes a stop action within a 1 m radius of the goal location is it considered to have navigated successfully. In addition, there must be no collisions and no loss during the continuous movement. According to [56], the SPL takes into account the efficiency of the robot in reaching the goal; for detailed descriptions, see Appendix A.

### 6.2. Evaluation on Simulation Data

**Figure 18:**
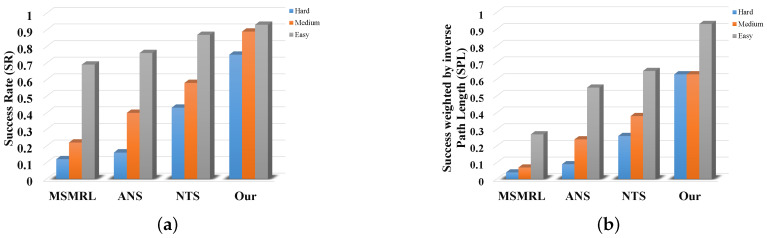
SRs and SPLs results for MSMRL, ANS, NTS, and our method on simulation scenarios. (**a**) Results of SRs; (**b**) Results of SPLs.

**Figure 19:**
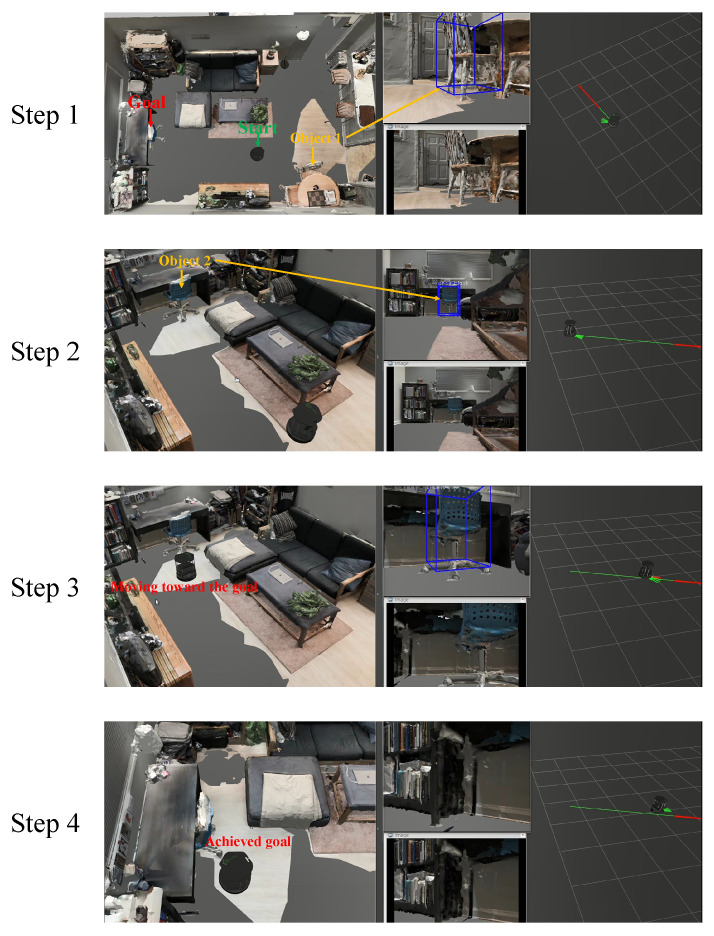
Visualization results for navigation in simulation scenario. The first step acquires global topological paths through active visual perception and object guidance, and generates segmented smooth trajectories. The second step determines the goal position by goal object evaluation and it generates segmented smooth trajectories. The third step moves toward the target location. The fourth step moves to within 1 meter of the goal object and publishes an "arrived" command to end the navigation.

We first compare the SR and SPL of our method with those of NTS [5], ANS [6], and MSMRL [7] on simulation data. The experimental results are shown in Table 1. Their bar graph representations of SR and SPL in Table 1 are shown in Figure 18a,b, respectively. It can be seen that our method outperforms all methods in all scenarios in terms of both SR and SPL. In addition, the performance improvements of our approach over the baseline increase along with the difficulty of experimental settings (0.06/0.05, 0.31/0.25, and 0.32/0.37). Our proposed method outperforms the MSMRL and ANS because of two potential reasons. First, we perform efficient and accurate object-based heuristic graph search to select paths instead of the image matching-based path retrieval method. The end-to-end path searching used by MSMRL and ANS has high computational complexity, especially in the large-scale environment. Second, compared to the metric map-based MSMRL and ANS, our proposed 3D environment modeling approach provides more reliable semantic–geometric features for navigation. The proposed trajectory generation and refinement strategies also improve the effectiveness of visual navigation. We can also see from Table 1 and Figure 18 that our method has better performance than NTS. It is mainly because the NTS is an image-based target-driven navigation method, in which the images do not provide directional information that can be explored for navigation [5], while our method provides multi-attribute information for navigation including the direction and pose of the object. In conclusion, these results of simulation experiments demonstrate the effectiveness of our proposed system. Figure 19 shows an example of our navigation visualization.

### 6.3. Evaluation on Real-World Data

**Figure 20:**
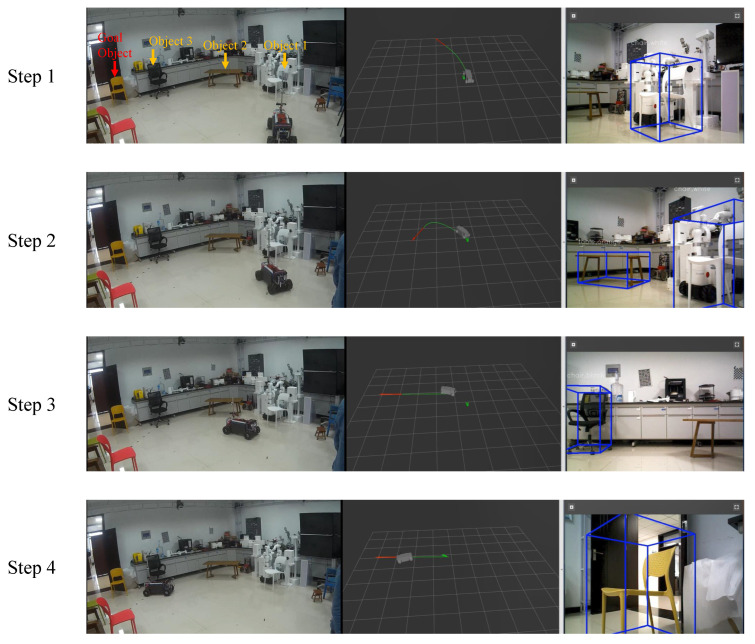
Visualization results for navigation in the S1 scene. We select an object-level topological path after constructing the lightweight topological semantic map. Then, the robot is guided from step one to step four to generate a segmented smooth trajectory to the next invisible object based on the currently visible object until it moves within 1 meter of the goal object and publishes an “arrived” command to end the navigation.

We further evaluate the performance of our method by comparing it with the well-known OGMADWA method in the real-world environment. Figure 16 visualizes the navigation environment. T1, T2, and T3 in Figure 16 correspond to the topological semantic maps of the three scenes, respectively, which are generated by our previous work [13]. G1, G2, and G3 in Figure 16 correspond to the high-precision occupancy grid maps of the navigation tasks respectively, which are generated by the OGMADWA method. Figure 20 shows the results of the visualization with the example of navigation in the S1 scene. We still choose SR and SPL as the evaluation metrics in the real-world experiment. For all experimental results, we report the median of 20 times.

**Figure 21:**
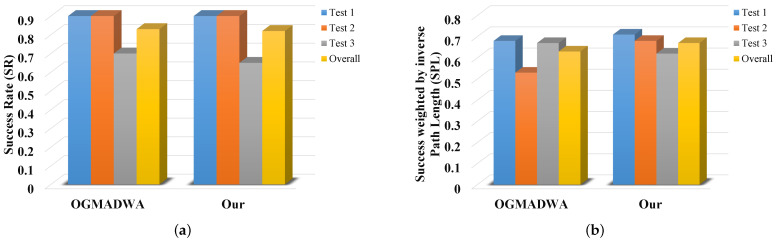
SRs and SPLs result for OGMADWA and our method on real-world datasets. (**a**) Results of SRs; (**b**) Results of SPLs.

The SR and SPL are shown in Table 2 and Figure 21. According to [56], the value of SPL greater than 0.5 indicates good navigation performance. It can be seen from Table 2 and Figure 21 that the SPL of both methods are greater than 0.5, which indicates that both methods have good navigation performance. We can still observe that our method outperforms the OGMADWA method. For example, as can be seen from the “Overall” column in Table 2, our method has similar SR with the OGMADWA method but has higher SPL than OGMADWA. This demonstrates the effectiveness and advantages of the proposed navigation method, since we do not need precise metric maps. We can further concretely observe the superiority of our method. In Test 1 and challenging Test 2, these two methods have the same SR, but our method has better performance in terms of SPL. There are two reasons. On one hand, we perform object-based topological path planning instead of prise metric map-based path planning. For the OGMADWAW method, when it comes to the multiple objects in S1 and dynamic objects in S2, the accuracy of the metric map decreases, and the complexity of the path planning method increases. Thus, its navigation performance decreases. As for our method, the proposed topological semantic map uses the semantic–geometric information and topological associations of objects, which has better scalability. On the other hand, our proposed object semantic–geometric feature-based heuristic evaluation strategy and Bernstein’s polynomial trajectory refinement method improve the efficiency of visual navigation. Particularly, unlike the A* algorithm, the proposed object-based heuristic path search method avoids over-dependence on high-precision metric maps. It is worth noting that for Test 3, we successfully navigate in a 26.2 m long weakly textured corridor, as shown in Figure 16S3. It can be seen that both methods achieve similar navigation performance, while OGMADWA achieves slightly higher on SR/SPL than our method. It is mainly because there are fewer available objects from the starting location to the goal location, which greatly challenges our method.

In summary, the above results show that the proposed method has a better overall performance compared to OGMADWA for the same configuration.

### 6.4. Discussion

From the above results, we have thoroughly verified the advantages of object-based global path planning. Then, for some special cases, we can discuss the limitations of our current approach. We represent the environment as an abstract topological graph in which the topological node and topological edge represent object-level landmarks and the semantic association properties between object-level landmarks, respectively. We model the environment mainly based on long static object-level landmarks. If the object in the model is dynamic and movable, then the topological relationship between the currently moving object and its neighbors changes, and the robot is at risk of being lost.

**Figure 22:**
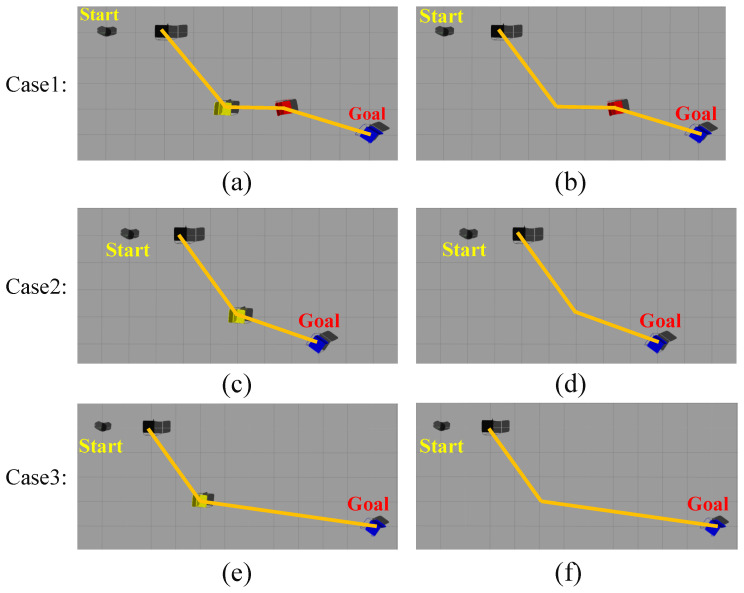
Navigation examples when objects in the environment models are moved. Yellow lines indicate the topological associations between objects. Case 1 shows that the robot can re-navigate through the surrounding red chairs after moving the yellow chair. The difference between Case 2 and Case 3 is that the distances between the goal object (blue chair) and the object being moved (yellow chair) are different. Case 3 shows that there is no detectable object around the robot and there is a risk of navigation failure after moving the yellow chair. (**a**,**c**,**e**) indicate the scenes before moving the yellow chair. (**b**,**d**,**f**) indicate the scenes after moving the yellow chair.

**Figure 23:**
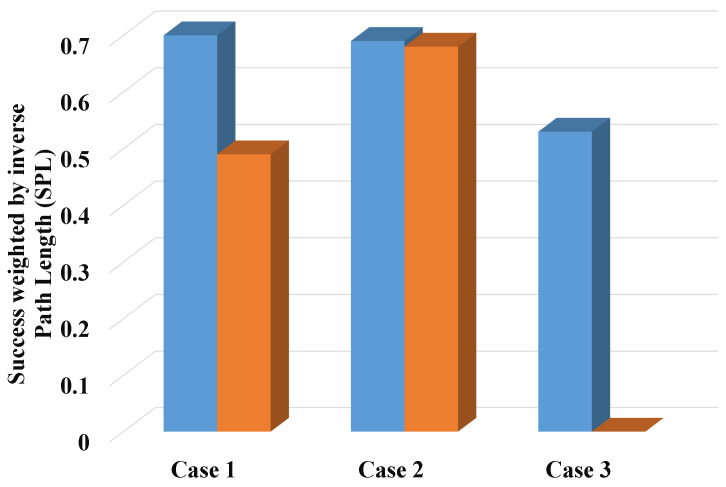
Bar graph of SPL results for all scenarios.

To effectively discuss and analyze the above question, we set up three cases to carry out navigation experiments, as shown in Figure 22. Case 1 shows that the robot can re-navigate through the surrounding red chairs after moving the yellow chair. The difference between Case 2 and Case 3 is that the distances between the goal object (blue chair) and the object being moved (yellow chair) are different. Case 3 shows that there is no detectable object around the robot and there is a risk of navigation failure after moving the yellow chair. Figure (a), Figure (c), and Figure (e) in Figure 22 indicate scenes before moving the yellow chair. Figure (b), Figure (d), and Figure (f) in Figure 22 indicate scenes after moving the yellow chair. We performed navigation experiments in these three cases and statistically calculated the SR and SPL of navigation respectively, as shown in Table 3. Their bar graph representations of SPL in Table 3 are shown in Figure 23. It can be seen from the experimental results that in Case 1 and Case 3, when the goal object in the map is moved, the SR and SPL of navigation decrease. In Case 1, although the topological relationship between the yellow chair and its neighboring objects disappears, the remain objects still maintain the original topological relationship well. Therefore, the robot can re-plan paths and regenerate a trajectory to the location of the goal object by searching for the surrounding red chair or goal object. In Case 2, when observable objects exist around the goal object, the robot can replan and re-navigate by searching for surrounding objects. In Case 3, when no observable objects exist around the goal object at all, the robot is lost, just as humans become lost. For the above problem, to perform effective navigation, we set five states for the robot, including initialization, waiting for the goal object, regenerating the trajectory, re-planning the path, and moving. The robot can online switch states according to the actual navigation situation.

## 7. Conclusions and Future Work

In this paper, we propose an object-based visual navigation method that can greatly improve navigation performance under the large-scale challenging environment. Firstly, a novel object-level semantic topological map is constructed to model the environment in a lightweight way. Then, we propose an object-based heuristic graph based path-searching method to obtain an optimal global topological path by combining semantic–geometric feature-based heuristic evaluation and topological association-based weight constraints. After that, to reduce the cumulative error of global navigation, active visual perception and object guidance are combined to segment global topological paths. Finally, we propose a new Bernstein polynomial refinement strategy to generate smooth and feasible navigation trajectories by parameterizing segmented paths. Experiments on 3D object feature detection in complex real-world scenes such as illumination changes and partial occlusions verify the high robustness of the proposed method. In addition, the effectiveness and reliability of the proposed global path planning method is verified by cumulative translation error and computational time cost experiments. The reliability of the proposed method is further proved by comparison experiments with SR and SPL of state-of-the-art methods under simulation and real-world scenarios. Despite the great results, some promotions are needed in the future. We will use a lightweight object detection network to improve the accuracy and reduce the computational complexity of the system as well as deploy the system on an embedded platform. We will also continue to improve the robot’s navigation performance in the case of dynamic changes in relationships between objects. Furthermore, we will explore the proposed method in more large-scale real-world navigation tasks.

## Figures and Tables

**Table 1:** Performance comparison results of the proposed algorithm with MSMRL, ANS, and NTS on the simulation datasets.

Model	Easy		Medium		Hard		Overall	
	SR	SPL	SR	SPL	SR	SPL	SR	SPL
MSMRL [7]	0.69	0.27	0.22	0.07	0.12	0.04	0.34	0.13
ANS [6]	0.76	0.55	0.40	0.24	0.16	0.09	0.44	0.29
NTS [5]	0.87	0.65	0.58	0.38	0.43	0.26	0.63	0.43
Our	**0.93**	**0.70**	**0.89**	**0.63**	**0.75**	**0.63**	**0.86**	**0.65**

**Table 2:** Performance comparison results of the proposed algorithm with OGMADWA on the real-world datasets.

Model	Test 1		Test 2		Test 3		Overall	
	SR	SPL	SR	SPL	SR	SPL	SR	SPL
OGMADWA	0.90	0.68	0.90	0.53	**0.70**	**0.67**	**0.83**	0.63
Our	**0.90**	**0.71**	**0.90**	**0.68**	0.65	0.62	0.82	**0.67**

**Table 3:** The SR and SPL of navigation in all scenarios.

Model	Case 1		Case 2		Case 3	
	(a)	(b)	(c)	(d)	(e)	(f)
SR	0.90	0.70	1	1	0.70	0
SPL	**0.70**	0.49	**0.69**	0.68	**0.53**	0

## Data Availability

The data presented in this study are available on request from the two corresponding authors. The data are not publicly available due to privacy restrictions.

## Appendix A. SPL

Success weighted by inverse Path Length (SPL) measures the robot’s navigation performance as

(A1) $$\frac{1}{N}\sum_{i=0}^{N}\;S_{i}\frac{l_{i}}{max(p_{i},l_{i})}$$

where *N* denotes the number of testing episodes, $S_{i}$ is a binary indicator of success in the testing episode *i*, $p_{i}$ represents path length, and $l_{i}$ is the shortest path distance from to the goal.

## References

[1] Zhang C., Liu Y., Wang F., Xia Y., Zhang W. (2018). Vins-mkf: A tightly-coupled multi-keyframe visual-inertial odometry for accurate and robust state estimation. Sensors.

[2] Milstein A. (2008). Occupancy grid maps for localization and mapping. Motion Plan.

[3] Liu Y., Bao L., Zhang C., Zhang W., Xia Y. Accurate and robust RGB-D dense mapping with inertial fusion and deformation-graph optimization. Proceedings of the 2019 IEEE 31st International Conference on Tools with Artificial Intelligence (ICTAI).

[4] Cadena C., Carlone L., Carrillo H., Latif Y., Scaramuzza D., Neira J., Reid I., Leonard J.J. (2016). Past, present, and future of simultaneous localization and mapping: Toward the robust-perception age. IEEE Trans. Robot..

[5] Chaplot D.S., Salakhutdinov R., Gupta A., Gupta S. Neural topological slam for visual navigation. Proceedings of the IEEE/CVF Conference on Computer Vision and Pattern Recognition.

[6] Chen T., Gupta S., Gupta A. (2019). Learning exploration policies for navigation. arXiv.

[7] Chaplot D.S., Gandhi D., Gupta S., Gupta A., Salakhutdinov R. (2020). Learning to explore using active neural slam. arXiv.

[8] Garg S., Sünderhauf N., Dayoub F., Morrison D., Cosgun A., Carneiro G., Wu Q., Chin T.J., Reid I., Gould S. (2021). Semantics for robotic mapping, perception and interaction: A survey. arXiv.

[9] Sun N., Yang E., Corney J., Chen Y. (2019). Semantic path planning for indoor navigation and household tasks. Annual Conference towards Autonomous Robotic Systems.

[10] Vasilopoulos V., Pavlakos G., Bowman S.L., Caporale J., Daniilidis K., Pappas G.J., Koditschek D.E. (2020). Technical report: Reactive semantic planning in unexplored semantic environments using deep perceptual feedback. arXiv.

[11] Klaas T., Lambrecht J., Funk E. Semantic Local Planning for Mobile Robots through Path Optimization Services on the Edge: A Scenario-based Evaluation. Proceedings of the 2020 25th IEEE International Conference on Emerging Technologies and Factory Automation (ETFA).

[12] Tang L., Wang Y., Ding X., Yin H., Xiong R., Huang S. (2019). Topological local-metric framework for mobile robots navigation: A long term perspective. Auton. Robot..

[13] Wang F., Zhang C., Tang F., Jiang H., Wu Y., Liu Y. (2022). Lightweight Object-level Topological Semantic Mapping and Long-term Global Localization based on Graph Matching. arXiv.

[14] Gao F., Wu W., Lin Y., Shen S. Online safe trajectory generation for quadrotors using fast marching method and bernstein basis polynomial. Proceedings of the 2018 IEEE International Conference on Robotics and Automation (ICRA).

[15] Kielas-Jensen C., Cichella V. BeBOT: Bernstein polynomial toolkit for trajectory generation. Proceedings of the 2019 IEEE/RSJ International Conference on Intelligent Robots and Systems (IROS), The Venetian Macao.

[16] Campos C., Elvira R., Rodríguez J.J.G., Montiel J.M., Tardós J.D. (2021). ORB-SLAM3: An Accurate Open-Source Library for Visual, Visual–Inertial, and Multimap SLAM. IEEE Trans. Robot..

[17] Wei H., Tang F., Xu Z., Zhang C., Wu Y. (2021). A Point-Line VIO System With Novel Feature Hybrids and with Novel Line Predicting-Matching. IEEE Robot. Autom. Lett..

[18] Wang F., Zhang C., Zhang G., Liu Y., Xia Y., Yang X. PLMCVIO: Point-Line based Multi-Camera Visual Inertial Odometry. Proceedings of the 2021 IEEE 11th Annual International Conference on CYBER Technology in Automation, Control, and Intelligent Systems (CYBER).

[19] Liu Y., Miura J. (2021). RDMO-SLAM: Real-time visual SLAM for dynamic environments using semantic label prediction with optical flow. IEEE Access.

[20] Fan Y., Zhang Q., Tang Y., Liu S., Han H. (2022). Blitz-SLAM: A semantic SLAM in dynamic environments. Pattern Recognit..

[21] Yang S., Scherer S. (2018). CubeSLAM: Monocular 3D object detection and SLAM without prior models. arXiv.

[22] Salas-Moreno R.F., Newcombe R.A., Strasdat H., Kelly P.H., Davison A.J. Slam++: Simultaneous localisation and mapping at the level of objects. Proceedings of the IEEE Conference on Computer Vision and Pattern Recognition.

[23] Marinakis D., Dudek G. (2010). Pure topological mapping in mobile robotics. IEEE Trans. Robot..

[24] Lui W.L.D., Jarvis R. A pure vision-based approach to topological SLAM. Proceedings of the 2010 IEEE/RSJ International Conference on Intelligent Robots and Systems.

[25] Labbé M., Michaud F. (2018). Long-term online multi-session graph-based SPLAM with memory management. Auton. Robot..

[26] Choset H., Nagatani K. (2001). Topological simultaneous localization and mapping (SLAM): Toward exact localization without explicit localization. IEEE Trans. Robot. Autom..

[27] Liu Y., Petillot Y., Lane D., Wang S. Global localization with object-level semantics and topology. Proceedings of the 2019 International Conference on Robotics and Automation (ICRA).

[28] Sánchez-Ibáñez J.R., Pérez-del-Pulgar C.J., García-Cerezo A. (2021). Path Planning for Autonomous Mobile Robots: A Review. Sensors.

[29] Elbanhawi M., Simic M. (2014). Sampling-based robot motion planning: A review. IEEE Access.

[30] Lavalle S.M. (1998). Rapidly-exploring random trees: A new tool for path planning. Comput. Sci. Dept. Oct..

[31] Kuffner J.J., LaValle S.M. RRT-connect: An efficient approach to single-query path planning. Proceedings of the IEEE International Conference on Robotics and Automation, Symposia Proceedings (Cat. No. 00CH37065).

[32] Karaman S., Frazzoli E. (2011). Sampling-based algorithms for optimal motion planning. Int. J. Robot. Res..

[33] Kavraki L.E., Svestka P., Latombe J.C., Overmars M.H. (1996). Probabilistic roadmaps for path planning in high-dimensional configuration spaces. IEEE Trans. Robot. Autom..

[34] Valero A., Gómez J.V., Garrido S., Moreno L. (2013). Fast Marching Method for safer, more efficient mobile robot trajectories. IEEE Robot. Autom. Mag..

[35] Noreen I., Khan A., Habib Z. (2016). Optimal path planning using RRT* based approaches: A survey and future directions. Int. J. Adv. Comput. Sci. Appl..

[36] Dijkstra E.W. (1959). A note on two problems in connexion with graphs. Numer. Math..

[37] Hart P.E., Nilsson N.J., Raphael B. (1968). A formal basis for the heuristic determination of minimum cost paths. IEEE Trans. Syst. Sci. Cybern..

[38] Stentz A. (1997). Optimal and efficient path planning for partially known environments. Intelligent Unmanned Ground Vehicles.

[39] Koenig S., Likhachev M., Furcy D. (2004). Lifelong planning A*. Artif. Intell..

[40] Dolgov D., Thrun S., Montemerlo M., Diebel J. (2010). Path planning for autonomous vehicles in unknown semi-structured environments. Int. J. Robot. Res..

[41] Ratliff N., Zucker M., Bagnell J.A., Srinivasa S. CHOMP: Gradient optimization techniques for efficient motion planning. Proceedings of the 2009 IEEE International Conference on Robotics and Automation.

[42] Berg J., Patil S., Alterovitz R. (2017). Motion planning under uncertainty using differential dynamic programming in belief space. Robotics Research.

[43] Tang S., Thomas J., Kumar V. (2018). Hold or take optimal plan (hoop): A quadratic programming approach to multi-robot trajectory generation. Int. J. Robot. Res..

[44] Mellinger D., Kumar V. Minimum snap trajectory generation and control for quadrotors. Proceedings of the 2011 IEEE International Conference on Robotics and Automation.

[45] Savkin A.V., Hoy M. (2013). Reactive and the shortest path navigation of a wheeled mobile robot in cluttered environments. Robotica.

[46] Zhou B., Pan J., Gao F., Shen S. (2021). Raptor: Robust and perception-aware trajectory replanning for quadrotor fast flight. IEEE Trans. Robot..

[47] Kielas-Jensen C., Cichella V. (2020). Bernstein polynomial-based transcription method for solving optimal trajectory generation problems. arXiv.

[48] Ding Z., Han X., Niethammer M. VoteNet: A deep learning label fusion method for multi-atlas segmentation. Proceedings of the International Conference on Medical Image Computing and Computer-Assisted Intervention.

[49] Guo Y., Wang H., Hu Q., Liu H., Liu L., Bennamoun M. (2020). Deep learning for 3d point clouds: A survey. IEEE Trans. Pattern Anal. Mach. Intell..

[50] Dai A., Chang A.X., Savva M., Halber M., Funkhouser T., Nießner M. Scannet: Richly-annotated 3d reconstructions of indoor scenes. Proceedings of the IEEE Conference on Computer Vision and Pattern Recognition.

[51] Song S., Lichtenberg S.P., Xiao J. Sun rgb-d: A rgb-d scene understanding benchmark suite. Proceedings of the IEEE conference on computer vision and pattern recognition.

[52] Smeulders A.W., Worring M., Santini S., Gupta A., Jain R. (2000). Content-based image retrieval at the end of the early years. IEEE Trans. Pattern Anal. Mach. Intell..

[53] Wolfe W.J., Mathis D., Sklair C.W., Magee M. (1991). The perspective view of three points. IEEE Trans. Pattern Anal. Mach. Intell..

[54] Xia F., Zamir A.R., He Z., Sax A., Malik J., Savarese S. Gibson env: Real-world perception for embodied agents. Proceedings of the IEEE Conference on Computer Vision and Pattern Recognition.

[55] Fox D., Burgard W., Thrun S. (1997). The dynamic window approach to collision avoidance. IEEE Robot. Autom. Mag..

[56] Batra D., Gokaslan A., Kembhavi A., Maksymets O., Mottaghi R., Savva M., Toshev A., Wijmans E. (2020). Objectnav revisited: On evaluation of embodied agents navigating to objects. arXiv.

